# Assessment of knowledge, attitudes and practices toward prevention of hepatitis B virus infection among students of medicine and health sciences in Northwest Ethiopia

**DOI:** 10.1186/s13104-016-2216-y

**Published:** 2016-08-19

**Authors:** Abdnur Abdela, Berhanu Woldu, Kassahun Haile, Biniam Mathewos, Tekalign Deressa

**Affiliations:** College of Medicine and Health Sciences, School of Biomedical and Laboratory Sciences, University of Gondar, P.O.Box 196, Gondar, Ethiopia

**Keywords:** Hepatitis B, Knowledge, Attitude, Occupational exposure, Health science students, Northwest Ethiopia

## Abstract

**Background:**

Hepatitis B virus (HBV) infection in the health setting is a global public health problem. The risk of occupational exposure to HBV among health care workers is a major concern, especially among students in health professions. In Ethiopia, very little is known about the knowledge, attitude, and practices (KAP) of trainees in the health professions towards occupational risk of HBV. Thus, the aim of this study was to assess the level of KAP of medicine and health Sciences students in Northwest Ethiopia towards occupational risk of HBV infection.

**Methods:**

A cross-sectional study was conducted from February 2015 to June 2015. A total of 246 students of health care professions were included into the study using a systematic random sampling technique. Data were collected using self-administered structured questionnaire and analysed by using SPSS version 20.

**Results:**

Majority of the study participants, (>80 %) had an adequate knowledge on risk factors for HBV, its mode of transmissions, and preventions. Two hundred of 246 (83.3 %) participants had positive attitude towards following infection control guidelines, and 201 (81.7 %) respondents believe that all HCWs should take HBV vaccine. However, only 5 (2 %) students had completed the three doses schedule of HBV vaccination. Whereas, a significant number of students, 66 (26.8 %), had been exposed to blood/body fluid via needle stick injury at least once since they started their training in the health facility.

**Conclusions:**

Our study found that trainees in health profession are at a very high risk of contracting HBV infection during their training owing to the low HBV vaccine uptake rate and high rate of accidental exposure to blood. Thus, we recommend that all students in the health profession should be vaccinated prior to their entry into professional practices.

**Electronic supplementary material:**

The online version of this article (doi:10.1186/s13104-016-2216-y) contains supplementary material, which is available to authorized users.

## Background

Hepatitis B virus (HBV) infection is one of the major public health problems in the world. According to the recent estimates, about one-third of the world population is infected with HBV [1, 2]. Of these, about 360 million people are chronic carriers and are at risk of developing liver diseases like cirrhosis and hepatocellular carcinoma (HCC). The prevalence of chronic HBV infection greatly varies worldwide (0.5–20 %), due to differences in age at the time of infection and mode of acquisition. A disproportionately high prevalence of chronic HBV infections exists in Southeast Asian and sub-Sahara African countries [3–5].

Health care workers (HCWs) are at high risk of HBV infection in the health care settings. The prevalence rate of HBV in HCWs is about 2–10 times higher than the general populations in the world [6, 7]. In context of HCWs, the risk factors for HBV infection are percutaneous or mucosal exposure to infected blood or body fluids, using inadequately sterilized medical equipment or contact with non-intact skin [7]. The average risk for acquiring HBV infection after percutaneous exposure to infected blood has been estimated to be 6–30 %; whereas it is about 0.3 % for human immunodeficiency virus [8].

The risk of occupational exposure to HBV infection is highly prevalent among HCWs in developing countries, where the prevalence of HBV is high in general population, and the health settings are poor [9–12]. A cross-sectional study conducted in Ethiopia, for instance, showed that 7.3 % of HCWs were infected with HBV while only 0.9 % of non-HCWs who participated in the study were infected [11]. Other studies conducted among medical waste handlers and blood donors in Ethiopia revealed an HBV infection rate of about 6 and 2.1 % respectively [12, 13]. The higher risk of HBV infection among HCWs in developing countries could be attributed to the prevailing careless handling of contaminated objects, reuses of inadequately sterilized medical equipment, and an improper waste disposal system [14, 15].

Apart from the HCWs, trainees in the health care professions are also exposed to an equal magnitude of occupational risk of HBV, as they work in the same health care delivery system. In fact, the risk for accidental exposure among the trainees could be higher due to their lack of experience, insufficient training, duty overload, and fatigue [16, 17].

Hepatitis B virus infection can be prevented by adhering to universal precautions including the use of protective barriers like gloves, proper sterilization of medical equipment, proper hospital wastes management system and vaccination [18–21]. Moreover, post-exposure prophylaxis can be used as a means of HBV prevention after accidental exposure to contaminated blood or body fluids [18–20]. However, studies have indicated that there is a clear gap of knowledge among trainees of health profession towards the risks of occupational exposure to HBV infection. A study from Lao democratic People’s Republic (Lao DPR), for example, has indicated that 86.5 % of medical students had poor knowledge on modes of HBV transmission and risk perception [22]. A similar study from Cameroon has indicated poor practice among the study participants, with only 10 % vaccination rate against HBV, and 55.9 % accidental exposure to blood [23].

Knowledge, attitude, and practices study measures key knowledge, feelings, tendencies, or skills commonly shared by a community on particular issues. It has been used as a useful study tool to design public health policies by taking into account the awareness, beliefs, and health seeking behavior of the at-risk population. In Ethiopia, data regarding knowledge, as well as attitude and practice towards the occupational exposure to HBV among the students of health care professions is scarce, albeit the high prevalence of the infection in the general population [24–26]. Therefore, the aim of this study was to assess the KAP of the students of medicine and health sciences towards HBV in Northwest Ethiopia.

## Methods

### Study site and population

This study was conducted at the college of medicine and health sciences, University of Gondar. The university is located in North Gondar Zone of the Amhara regional state, Northwest Ethiopia. The College of medicine and health sciences is one of the oldest medical education institutions in the country. It has a teaching hospital, and different schools including the school of medicine, pharmacy, biomedical and laboratory sciences, institutes of public health, and various departments. Most of the health care profession students receive 4 years training except for the students of medicine (6 years) and Pharmacy (5 years). The study population was students of medicine and health sciences who had clinical attachments for a minimum of about 2 years.

### Study design and period

A cross-sectional study was conducted to assess KAP of the fifth year medical and fourth year health science students toward prevention of HBV infection. The study was conducted from February 2015 to June 2015.

### Definitions for scoring knowledge, attitude and practices

The following operational definitions were used in this study. Good knowledge: if the respondents were able to answer 70 % or more of knowledge items correctly. Poor knowledge: if the respondents answered less than 70 % of knowledge items. Positive attitude: if the respondents were are able to give the correct answer for 70 % or more of attitude items. Negative attitude: if the respondents answered less than 70 % of attitude items. Good practice: when the study participants were at least able to answer 70 % or more practice items correctly. Malpractice: when the participants were unable to answer 70 % of practice items correctly.

### Sample size determination

A single population proportion formula was used to estimate sample size. Since there was no similar study from Ethiopia, the following assumptions have been made: 95 % confidence interval (Z_α/2_ = 1.96), 50 % proportion, and 5 % margin of error.

$${\text{N}} = \frac{{(Z_{\alpha{/}2}) \times {\text{XP}}(1 - {\text{P}})}}{{d^{2} }},\quad {\text{N}} = \frac{{(1.96^{2} ) \times 0.5(1 - 0.5)}}{{(0.05)^{2} }} = 384$$

However, since the number of source population for the study was 535, which was less than 10,000, we used the following correction formula [27]:

$${\text{nf}} = {\text{ni}} / (1+{\text{ni/N}}), \quad {\text{nf}} = 384/1 + (384/535) = 223.55;$$

where nf = corrected sample size, ni = uncorrected sample size, and N = total number of all the source population.

By adding 10 % non-response rate, we included a total of 246 study subjects. The total sample size was distributed proportionally to each department based on their student population. The study participants were selected by a random sampling technique.

### Study variables

Knowledge, attitude, and practice of the study participants towards HBV transmission and prevention were considered as dependent variables; and sex, age, residence areas, and departments of the study population were considered as the independent variables.

### Data collection

A self-administered structured questionnaire was used to collect information about the socio-demographic characteristics of respondents, KAP towards transmission and prevention of HBV infection (see an Additional file 1).

### Statistical analysis

Data were entered, cleaned and analyzed using SPSS version 20 statistical package software (SPSS Inc., Chicago, IL). Descriptive statistics like frequencies and proportions were used to summarize the data. Bivariate and multivariate analyses were used to examine the relationship between the outcome variables (mean knowledge, attitude, and practice) and selected socio-demographic factors. Adjusted odds ratios (AOR) and their 95 % confidence intervals (CIs) were used as indicators of the strength of association. Statistical significance was set at P values of less than 0.05.

## Results

### Characteristics of the study participants

A total of 246 students belonging to nine departments were approached for the study. All of them were participated in the study making a response rate of 100 %. The majority of the students 217 (88.2 %) were in the age group of 20–24 years and 187 (76 %) were male. A little more than half 144 (58.5 %) of the participants were from urban areas (Table 1).

**Table 1:** Baseline characteristics of the study participants at the college of medicine and health sciences, University of Gondar, Northwest Ethiopia

Variables	N (%)
Gender	
Male	187 (76)
Female	59 (24)
Age	
20–24	217 (88.2)
25–29	29 (11.8)
Department	
Medicine	74 (30.1)
MLT	17 (6.9)
PHO	35 (14.2)
Physiotherapy	11 (4.5)
Nurse	34 (13.8)
Midwifery	33 (13.4)
Anaesthesia	12 (4.9)
Optometry	11 (4.5)
Psychiatry	19 (7.7)
Residence	
Rural	102 (41.5)
Urban	144 (58.5)

*MLT* medical laboratory technologists, *PHO* public health officer, *PHO* students receive training for 4 years with a focus on primary care, with minor surgical procedures, as well as managerial health care service. The PHO serves mostly in health centers

### Knowledge level of the respondents on HBV

Overall, most of the study participants had adequate knowledge on HBV infection and its mode of transmission. Of the students surveyed, 200 (81.3 %) knew that HBV infection associate with liver cancer. Regarding the mode of transmission, 239 (97.2 %) reported contact with blood or body fluid of HBV carriers, 238 (96.7 %) mentioned unsterilized medical equipment such as needle and syringes, and 207 (84.1 %) answered unsafe sexual contact (Table 2). In terms of knowledge on vaccination, 84.6 % of the respondents were aware of HBV vaccine and that it provides protection against HBV infection. However, relatively a low proportion (67.1 %) of study participants knew that HBV has a post-exposure prophylaxis and that it can be treated/or cured (52.4 %).

**Table 2:** Knowledge level of the students at the college of medicine and health science, University of Gondar, 2015

Hepatitis B knowledge questions	Yes n (%)	No n (%)	Not sure n (%)
HBV causes liver cancer	200 (81.3)	10 (4.1)	36 (14.6)
HBV carriers can transmit the infection	235 (95.5)	4 (1.6)	7 (2.8)
HBV spread by casual contact such as hand shacking	153 (62.2)	79 (32.1)	14 (5.7)
HBV spread by contact with open wounds/cut?	230 (93.5)	9 (3.7)	7 (2.8)
HBV can be transmitted by contaminated blood and body fluids	239 (97.2)	7 (2.8)	00 (0.0)
HBV can be transmitted by unsterilized syringes, needles and surgical instruments	238 (96.7)	8 (3.3)	00 (0.0)
Hepatitis B transmitted by unsafe sex	207 (84.1)	39 (15.9)	00 (0.0)
Vaccine can prevent hepatitis B infection	208 (84.6)	19 (7.7)	19 (7.7)
Do you think HBV has laboratory test?	238 (96.7)	8 (3.3)	00 (0.0)
HBV has post exposure prophylaxis	165 (67.1)	81 (32.9)	00 (0.0)
Hepatitis B can be cured/treated	129 (52.4)	117 (47.6)	00 (0.0)

### Attitudes towards HBV infection and risk perception

The attitudes of students of medicine and health sciences towards HBV infection are summarized in Table 3. About 77 % of the students were aware that they are at-risk for HBV infection, and 83.3 % agreed that following infection control guidelines would protect them from being infected at work. Further, 81.7 % of the students acknowledged that vaccine against HBV prevents getting the infection.

**Table 3:** Attitudes of the students of medicine and health sciences towards hepatitis B prevention, University of Gondar

Attitude questions	Agree N (%)	Disagree N (%)	Not sure N (%)
I have no concern of being infected with HBV	31 (12.6)	190 (77.2)	25 (10.2)
Hepatitis B vaccine is safe and effective	201 (81.7)	24 (9.8)	21 (8.5)
Changing of gloves during blood collection and tests is waste of time	14 (5.7)	223 (90.7)	9 (3.7)
All patients should be tested for HBV before they receive health care	137 (55.7)	91 (37.0)	18 (7.3)
I do not feel comfortable to take care of people with HBV	27 (11.0)	202 (82.1)	17 (6.9)
Following infection control guidelines will protect from being infected with HBV at work?	205 (83.3)	33 (13.4)	8 (3.3)

To assess their attitudes toward discrimination and stigma on HBV carriers, we asked whether they are comfortable in treating HBV patients. About 82 % (202/246) of the students had responded in agreement to the inquiry. On the other hand, 55.7 % of the students think that all patients need to be tested before receiving any health care services.

Multivariate analysis of the knowledge of trainees in the health care profession revealed that of those trainees at higher risk of HBV infection, students of nursing (AOR5.87, 95 % CI 1.05–32.88), midwifery (AOR 2.02, 95 % CI 0.26–15.21) and anesthesia (AOR 2.93, 95 % CI 0.24–35.99) had lower knowledge on HBV compared to the students of medicine. Further, of the at-risk groups, students of nursing (95 % CI 4.70–34.11), midwifery (95 % CI 4.51–33.68), and anesthesia (95 % CI 1.25–16.94) showed unfavorable attitude towards HBV prevention with the odds of 12.67, 12.32 and 4.61 respectively (Table 4).

**Table 4:** Multivariate analysis of factors associated with poor knowledge and attitudes towards hepatitis B prevention among medicine and health Science students at the University of Gondar, 2015

Variables	Knowledge		AOR (95 % CI)	P value	Attitude		AOR (95 % CI)	P value
	Good	Poor			Favorable	Not		
Sex								
Male	160 (85.6)	27 (14.4)	1.3 (0.5–3.8)	0.59	104 (55.6)	83 (44.4)	1.3 (0.6–2.7)	0.51
Female	52 (88.1)	7 (11.9)	1.0	–	30 (50.8)	29 (49.2)	1.0	
Age								
20–24	185 (85.3)	32 (14.7)	3.8 (0.6–23.3)	0.14	115 (53.0)	102 (47.0)	1.5 (0.5–3.9)	0.45
25–29	27 (93.1)	2 (6.9)	1.0	–	19 (65.5)	10 (34.5)	1.0	–
Residence								
Rural	86 (84.3)	16 (15.7)	1.2 (0.5–2.7)	0.71	54 (52.9)	48 (47.1)	0.8 (0.4–1.4)	0.41
Urban	126 (87.5)	18 (12.5)	1.0	–	80 (55.6)	64 (44.4)	1.0	–
Profession								
Medicine	72 (97.3)	2 (2.7)	1.0	–	60 (81.1)	14 (18.9)	1.0	–
MLT	17 (100)	0 (0.0)	0.0	0.99	10 (58.8)	7 (41.2)	2.7 (0.9–8.5)	0.09
PHO	26 (74.3)	9 (25.7)	10.9 (2.2–55.2)	0.00	27 (77.1)	8 (22.9)	1.3 (0.5–3.5)	0.63
Nursing	29 (85.3)	5 (14.7)	5.87 (1.1–32.9)	0.04	9 (26.5)	25 (73.5)	12.7 (4.7–34.1)	0.00
Midwifery	31 (93.9)	2 (6.1)	2.02 (0.3–15.5)	0.49	9 (27.3)	24 (72.7)	12.3 (4.5–33.7)	0.00
Anaesthesia	11 (91.7)	1 (8.3)	2.93 (0.2–36.0)	0.40	6 (50.0)	6 (50.0)	4.6 (1.3–16.9)	0.02
Physiotherapy	8 (72.8)	3 (27.3)	11.6 (1.7–82.2)	0.01	3 (27.3)	8 (72.7)	11.6 (2.7–50.3)	0.00
Optometry	4 (36.4)	7 (63.6)	81.7 (11.4–585)	0.00	2 (18.2)	9 (81.8)	22.5 (4.2–122.3)	0.00
Psychiatry	14 (73.7)	5 (26.3)	11.0 (1.9–63.4)	0.01	8 (42.1)	11 (57.9)	5.4 (1.8–16.1)	0.00
Overall	212 (86.2)	34 (13.8)	–	–	134 (54.5)	112 (45.5)	–	–

*MLT* medical laboratory technologists, *PHO* public health officer

### Practical measures for HBV prevention and Health seeking behavior

Of the 246 participants, only 23 (9.3 %) had screened for HBV, 12 (4.9 %) students had vaccinated against HBV, and only 5 (2 %) of vaccinated students had completed the recommended three doses. About 27 % of the respondents had a needle stick injury, and 53.7 % of the participants had responded that they would report if they had needle stick injury (Table 5). Overall, there were poor practical measures on prevention of HBV infection among the study subjects.

**Table 5:** Practices of medicine and health sciences students towards hepatitis B prevention at the University of Gondar hospital, 2015

HBV practice questions	Yes number (%)	No number (%)
Have you ever screened for hepatitis B?	23 (9.3)	223 (90.7)
Have you got vaccinated against HBV?	12 (4.9)	234 (95.1)
How many doses of HBV vaccine did you receive?		
One dose	6 (2.4)	–
Two doses	1 (0.4)	–
Three doses	5 (2.0)	–
I always change gloves for each patient during blood taking	209 (85.0)	37 (15.0)
Have you ever had a needle prick injury?	66 (26.8)	180 (73.2)
I always report for needle stick injury	132 (53.7)	114 (46.3)

## Discussions

Exposure to blood-borne pathogens such as HBV infection remains a significant occupational hazard to HCWs, especially in countries where this infection is highly prevalent. KAP surveys have been used as important sources of data to design health intervention methods and public health policies. In Ethiopia, there is a paucity of data regarding the knowledge and practices towards occupational hazard of HBV among trainees in the health profession. The current study describes the KAP towards HBV infection among medical and health science students at the University of Gondar.

Despite the wide professional background of the study participants, our results showed that overall knowledge regarding HBV, its mode of transmission and prevention was high (86.2 %). Most respondents knew that exposure to infected blood or body fluid, contaminated needles, contact with non-intact skin or unsafe sexual contacts are risk factors for HBV infection. This finding was consistent with the previous study from Cameroon that reported a good knowledge of the study participants on HBV infection [23]. But, it was higher than the 56.2 % knowledge levels at Haramaya University, Ethiopia [24], 59 % from Iraq [28] and 14.5 % from Lao DPR [22]. Nevertheless, we found that relatively lower proportion of the students knew that HBV has treatments (52.4 %) and post-exposure prophylaxis (67.1 %). This indicating that there is a need to alleviate the gaps as these might affect seeking medical attention.

In this study, the overall attitude towards HBV prevention among the participants was favorable. More than 75 % of the participants were aware that they are at risk of contracting HBV and believe that HBV vaccine is effective and safe. This finding was in line with the report from Saudi Arabia among dentists [29]. Our study revealed that most of the study participants had malpractice towards HBV prevention, in spite of their good knowledge and positive attitude on the disease and its prevention measures. Risky practices among the study participants were highly prevalent, with 28.6 % of them had exposed to blood accidentally; and 46.3 % had no intension to report the accident. This finding suggesting that there is a need to address the gap by strengthening health education on universal safety precaution for prevention of infections. Yet, when compared with the reports from other countries, the finding 28.6 % rate of accidental exposure to blood was lower than the 55.9 % rate reported from Cameroon [23], 48 % from Nigeria [30], and 40 % from Palestine [31].

In terms of vaccination against HBV, WHO recommends the preventive vaccine to all HCWs in countries with high HBV endemicity. However, this study revealed the least vaccine acceptance rate (2 %) among the study participants compared to a number of similar studies [22, 23, 32]. The low HBV vaccine acceptance rate among the study participants might be partly ascribed to the inaccessibility of HBV vaccine as it was included into the Ethiopian immunization program in 2007.

This study has a limitation in that we could not confirm the information regarding HBV vaccination as the study participants came from different parts of the country. The data was obtained by questionnaire, and therefore, there could be a recall bias of the participants. Despite the limitations, the findings of this study highlighted that there is a critical need for immunizing trainees in health profession against this highly contagious pathogen.

## Conclusions

Our data demonstrated that trainees in health profession are at a very high risk of contracting HBV infection during their training owing to low HBV vaccine uptake rate and high rate of accidental exposure to blood. Thus, we recommend that all students in the health care profession should be vaccinated prior to their entry into professional practices.

## Additional file

10.1186/s13104-016-2216-y A questionnaire used in this study to collect data on knowledge, attitude and practices of trainees in the health care profession towards HBV.

## Abbreviations

KAP: knowledge, attitude, and practices
HBV: hepatitis B virus
HCC: hepatocellular carcinoma
HCW: health care workers
AOR: adjusted odds ratios
CI: confidence intervals
MLT: medical laboratory technologists
PHO: public health officer
