# Supplementary material for: Assessment of knowledge, attitudes and practices toward prevention of hepatitis B virus infection among students of medicine and health sciences in Northwest Ethiopia
Source: BMC Res Notes. 2016 Aug 19;9:410. doi: 10.1186/s13104-016-2216-y (PMC4992214; doi:10.1186/s13104-016-2216-y)
Supplement: Supplementary file 1 — 10.1186/s13104-016-2216-y A questionnaire used in this study to collect data on knowledge, attitude and practices of trainees in the health care profession towards HBV. [file 13104_2016_2216_MOESM1_ESM.docx]

**Questionnaires for HBV related KAP Survey**

This questionnaire is designed to assess the knowledge, attitude and practices of trainees in the health care profession towards hepatitis B virus infection. Please take time to read and answer each question carefully by circling the best alternative that represents your response.

Please do not write your name.

Code:__________

| **Section A: Demographics** | |
| --- | --- |
| 1. Gender | 1. Male 2. Female |
| 2. Age category (years) | 1. 20-24 2. 25-29 3. >30 |
| 3.Indicate your department | 1.Medicine, 2.MLT, 3.PHO, 4.physiotherapy, 5. Nurse, 6. midwifery, 7. Anaesthesia, 8. Optometry and 9. Psychiatry |
| 4.Residence(your background residence) | 1. Rural 2. Urban |
| **Section B: Knowledge Items** | |
| 1. People who are carriers of hepatitis B are at risk of infecting others. | 1. Yes 2. No 3. Don’t know |
| 2. Can hepatitis B be caught through casual contact such as holding of hands? | 1. Yes 2. No 3. Don’t know |
| **3.** Can hepatitis B be spread through contact with open wounds/cuts? | 1. Yes 2. No 3. Don’t know |
| **4.** Can hepatitis B virus cause liver cancer? | 1. Yes 2. No 3.Don’t know |
| 5. Can HBV vaccine prevent Hepatitis B? | 1. Yes 2. No 3.Don’t know |
| 6**.** Can Hepatitis B be transmitted by contaminated blood and blood products? | 1. Yes 2. No |
| 7. Can Hepatitis B be transmitted by un-sterilized syringes, needles and surgical instruments? | 1. Yes 2. No |
| 8. Can Hepatitis B be transmitted by unsafe sex? | 1. Yes 2. No |
| 9. Do you think HBV has laboratory test? | 1. Yes 2. No |
| 10**.** Is Hepatitis B curable/treatable? | 1. Yes 2. No |
| 11. Do you think that HBV has post exposure prophylaxis? | 1. Yes 2. No |
| **Section C: Attitude Item Questions** | |
| 1**.** I am not at risk for getting hepatitis B | 1. Agree 2. Disagree 3. Not sure |
| 2. I do not believe in the hepatitis B vaccine | 1. Agree 2. Disagree 3. Not sure |
| 3. Changing of the gloves during blood collection and test is a waste of time | 1. Agree 2. Disagree 3. Not sure |
| 4. All patients should be tested for HBV before they receive health care | 1. Agree 2. Disagree 3. Don’t have any idea. |
| 5. I do not like treating people with HBV | 1. Agree 2. Disagree 3. Don’t have any idea. |
| 6. Following infection control guidelines will protect me from being infected with HBV at work. | 1. Agree 2. Disagree 3. Don’t have any idea. |
| **Section D: Practice Item Questions** | |
| 1. Have you done screening for Hepatitis B? | 1. Yes 2. No |
| 2. Have you got yourself vaccinated against Hepatitis B? | 1. Yes 2. No |
| 3. If your answer is yes in question#23How many doses of hepatitis B vaccine did you receive? | 1. One dose 2. Two doses  3. Three doses |
| 4. I always change gloves for each patient during blood taking | 1. Yes 2. No |
| 5. Have you ever had a needle prick injury? | 1. Yes 2. No |
| 6. I always report for a needle stick injury | 1. Yes 2. No |
